# OKlahoma Nitrone-007: novel treatment for diffuse intrinsic pontine glioma

**DOI:** 10.1186/s12967-020-02593-5

**Published:** 2020-11-10

**Authors:** Lincy Thomas, Nataliya Smith, Debra Saunders, Michelle Zalles, Rafal Gulej, Megan Lerner, Kar-Ming Fung, Angel M. Carcaboso, Rheal A. Towner

**Affiliations:** 1 Advanced Magnetic Resonance Center, Oklahoma Medical Research Foundation, 825 NE 13th Street, Oklahoma City, OK 73104 USA; 2 Department of Pathology, University of Oklahoma Health Sciences Center, Oklahoma City, OK USA; 3 The Jimmy Everest Center for Cancer and Blood Disorders in Children, University of Oklahoma Health Sciences Center, Oklahoma City, OK USA; 4 Oklahoma Center for Neuroscience, University of Oklahoma Health Sciences Center, Oklahoma City, OK USA; 5 Surgery Research Laboratory, University of Oklahoma Health Sciences Center, Oklahoma City, OK USA; 6 Stephenson Cancer Center, University of Oklahoma Health Sciences Center, Oklahoma City, OK USA; 7 Pharmaceutical Department, Medical University of Lodz, Lodz, Poland; 8 Department of Pediatric Hematology and Oncology, Hospital Sant Juan de Deu, Institut de Recerca Sant Joan de Deu, Barcelona, Spain; 9 Present Address: University of Texas Southwestern in the Division of Hematology and Oncology, Dallas, TX USA

**Keywords:** Diffuse intrinsic pontine glioma (DIPG), ACVR1 mutation, Patient-derived xenograft (PDX), OKN-007, Magnetic resonance imaging (MRI), Diffusion-weighted imaging (DWI), Microvessel density (MVD), Apoptosis, H3K27 trimethylation

## Abstract

**Background:**

Diffuse intrinsic pontine glioma (DIPG) is the most common brainstem cancer in childhood. This rapidly progressing brainstem glioma holds a very dismal prognosis with median survival of less than 1 year. Despite extensive research, no significant therapeutic advancements have been made to improve overall survival in DIPG patients.

**Methods:**

Here, we used an orthotopic xenograft pediatric DIPG (HSJD-DIPG-007) mouse model to monitor the effects of anti-cancer agent, OKlahoma Nitrone-007 (OKN-007), as an inhibitor of tumor growth after 28 days of treatment. Using magnetic resonance imaging (MRI), we confirmed the previously described efficacy of LDN-193189, a known activin A receptor, type I (ACVR1) inhibitor, in decreasing tumor burden and found that OKN-007 was equally efficacious.

**Results:**

After 28 days of treatment, the tumor volumes were significantly decreased in OKN-007 treated mice (p < 0.01). The apparent diffusion coefficient (ADC), as a measure of tissue structural alterations, was significantly decreased in OKN-007 treated tumor-bearing mice (p < 0.0001). Histological analysis also showed a significant decrease in CD34 expression, essential for angiogenesis, of OKN-007 treated mice (p < 0.05) compared to LDN-193189 treated mice. OKN-007-treated mice also significantly decreased protein expression of the human nuclear antigen (HNA) (p < 0.001), ACVR1 (p < 0.0001), and c-MET (p < 0.05), as well as significantly increased expression of cleaved caspase 3 (p < 0.001) and histone H3 K27-trimethylation (p < 0.01), compared to untreated mouse tumors.

**Conclusions:**

With the dismal prognosis and limited effective chemotherapy available for DIPG, there is significant room for continued research studies, and OKN-007 merits further exploration as a therapeutic agent.

## Background

Diffuse intrinsic pontine glioma (DIPG) is an invasive pontine tumor that affects approximately 300 children in the United States yearly [1] It is the most common brainstem tumor, and affected children are most commonly between the ages of 4 and 11 [2]. DIPG holds a grim prognosis with median survival less than 1 year [3]. Different therapeutic approaches have been attempted to target DIPG including chemotherapy (i.e. vincristine, lomustine), targeted monoclonal therapy (bevacizumab), involved field radiation therapy, chemotherapy in conjunction with radiation therapy, intraventricular chemotherapy. Despite these efforts, the clinical course and prognosis of DIPG has not changed significantly. Large clinical centers currently use involved field radiation in combination with chemotherapy, but no standard of care practices have been established at this time. The extremely poor prognosis is due to its aggressive biology, only a transient response to radiation, with ineffective response to chemotherapeutic agents delivered on its own, as DIPG has an intact blood brain barrier (BBB) [1]. Until recently, DIPG usually has been a diagnosis made by magnetic resonance imaging (MRI) findings alone. This is a great area of change as large clinical centers are now obtaining tissue biopsy at the time of diagnosis to better unravel the true biology of DIPG. The World Health Organization 2016 classification of central nervous system tumors replaces the clinical name of DIPG to Diffuse Midline Glioma, H3 K27M-mutant. *H3K27M* mutant gliomas are associated with worse clinical outcome and poor treatment response compared to wild-type tumors [4]. Approximately 80% of DIPG tumors have a histone H3 mutation, which results in the replacement of lysine 27 with methionine (K27M). This occurs in genes encoding histone H3 variant H3.3 (H3F3A) or H3.1 (HIST1H3B). H3.3-K27M increases expression of PDGFRA and Pax3. H3.1-K27M is usually a co-mutation with ACVR1 mutations, which is the second most common mutation in DIPG, with approximately 25% of cases harboring this mutation [5]. These mutations contribute to the aggressive biology and poor treatment response. Within the last decade, DIPG has had no significant improvement in overall survival, and there is clearly a dire need for therapeutic advancements [2].

The goal of our research is to assess the effectiveness of OKN-007 (OKlahoma Nitrone-007; disodium 4-[(tert-butyl-imino) methyl] benzene-1,3-disulfonate *N*-oxide) as a therapeutic agent for a pediatric DIPG mouse model. OKN-007 has shown promise in prior studies involving pediatric glioblastoma mouse models by decreasing growth of blood vessels, which is essential for any tumor progression [6]. G55 is a human adult glioblastoma brain tumor initiating cell (BTIC) line that is used to create xenograft glioma models. G55 glioma bearing mice had a statistically significant increase in percent survival and decreased tumor volumes in the OKN-007 treated arm compared to the untreated group [7]. OKN-007 is novel, as it overcomes one of the barriers of systemic chemotherapy administration, which is traversing of the BBB. With the effectiveness that OKN-007 has shown as an anti-cancer agent in preclinical models, it is currently undergoing clinical trial assessment as an investigational drug for recurrent adult glioblastomas, as well as newly diagnosed patients when combined with temozolomide [7–9]. OKN-OO7 has both anti-inflammatory and proapoptotic features. Immunohistochemistry (IHC) has shown the ability of OKN-007 to decrease cell proliferation by inhibiting glucose transporter 1 (GLUT-1), decrease angiogenesis measured by levels of CD-31 (an endothelial marker to assess microvascular density), decrease the expression of hypoxia inducible factor 1 α (HIF-1α) with reduced hypoxia (or increased oxygenation), and by increasing apoptosis (cleaved caspase-3) compared to untreated controls [9]. In human liver cancer cells, OKN-007 was noted to have an anti-tumor effect by suppression of both the transforming growth factor β1 (TGFβ1)/SMAD and Hedgehog/GLII signaling pathway by inhibiting sulfatase 2 (SULF2) enzyme activity [10]. SULF2 functions as an oncoprotein in hepatocellular carcinoma and treatment with OKN-007 significantly decreased SULF2 activity, which is associated with PDGFRα expression. OKN-007 treated F98 gliomas had substantially decreased TGFB1 protein levels, resulting in TGFB1 inhibition as the upstream regulator, and 57 downregulated genes within this pathway [7]. Molecular-targeted MRI probes were also used to show that OKN-007 can reduce protein expression of vascular endothelial growth factor receptor 2 (VEGFR2) and reduce free radical levels [11, 12]. Clearly, OKN-007 has antitumor effects against preclinical models of glioblastoma, and liver cancers, and it is worthwhile assessing if similar effects can be seen for a fatal diagnosis such as DIPG. In addition to OKN-007, we also evaluated the efficacy of a known ACVR1 inhibitor, LDN-193189. This agent was tested in a DIPG preclinical mouse model and demonstrated prolonged survival in treated mice compared to untreated, with immunohistochemistry confirming less human nuclear antigen positivity in treated mice [5].

We used a previously described in vivo DIPG model using post-mortem patient-derived neurospheres, HSJD-DIPG-007 [13, 14]. This specific cell line was used because it harbors both the *H3 K27M* and *ACVR1* mutation, creating the most aggressive pre-clinical model to assess the efficacy of OKN-007.

**Fig. 1:**
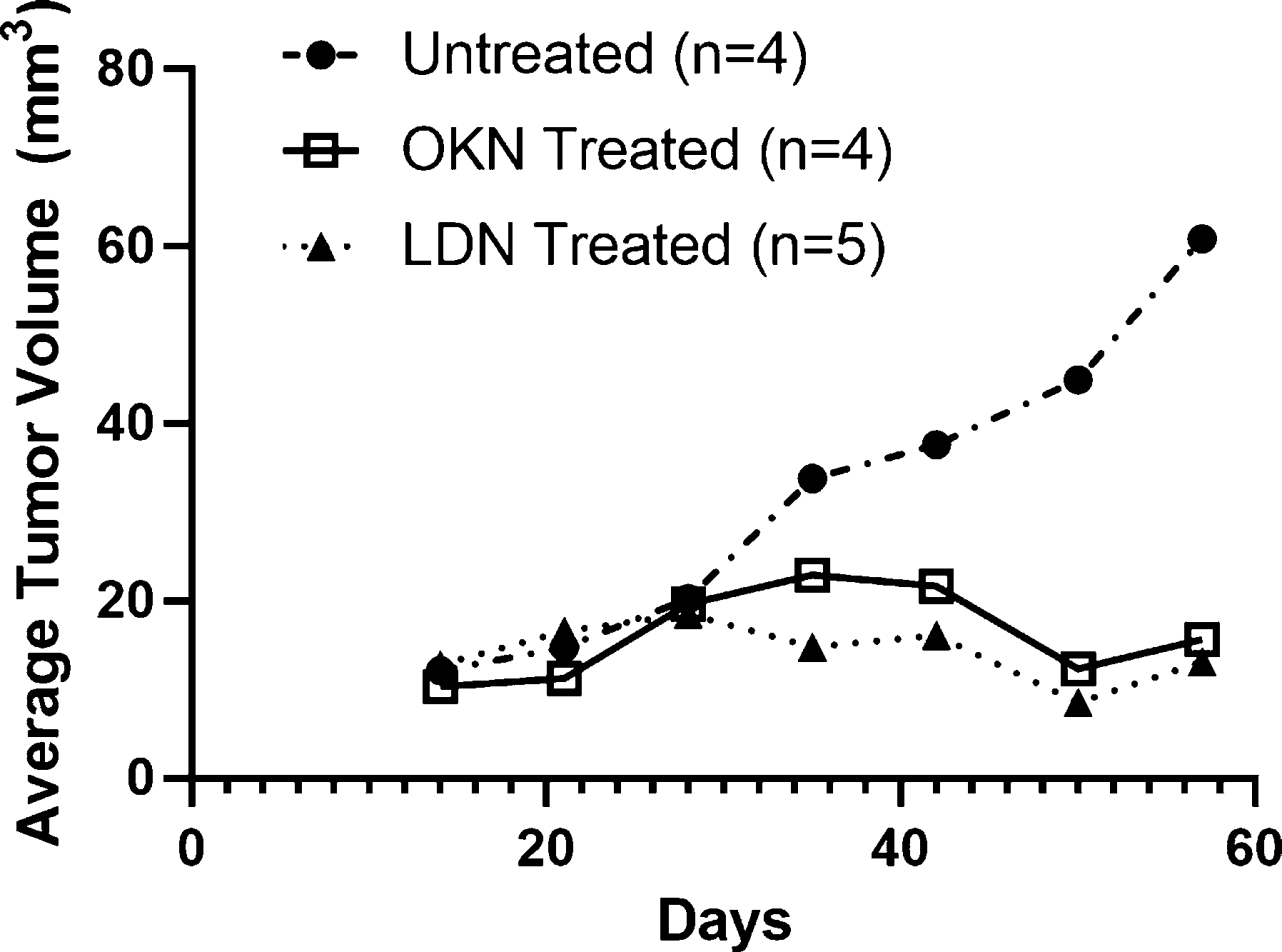
OKN-007 and LDN-193189 both decrease tumor volumes in a pre-clinical model for DIPG. Average weekly tumor volumes for each treatment arm. All three treatment groups had similar tumor volumes from day 14 to day 28, prior to treatment initiation. Once treatment started on day 28, there is clear divergence of tumor volumes between each treatment group. Tumor volumes for both OKN-007 (open squares with solid line) and LDN-193189 (closed triangles with dotted line) treated mice had stabilized over time. Unlike the final tumor volume in the untreated mice (closed circle with dot-dash line), which was notably higher than mice treated with both OKN-007 and LDN-193189

**Fig. 2:**
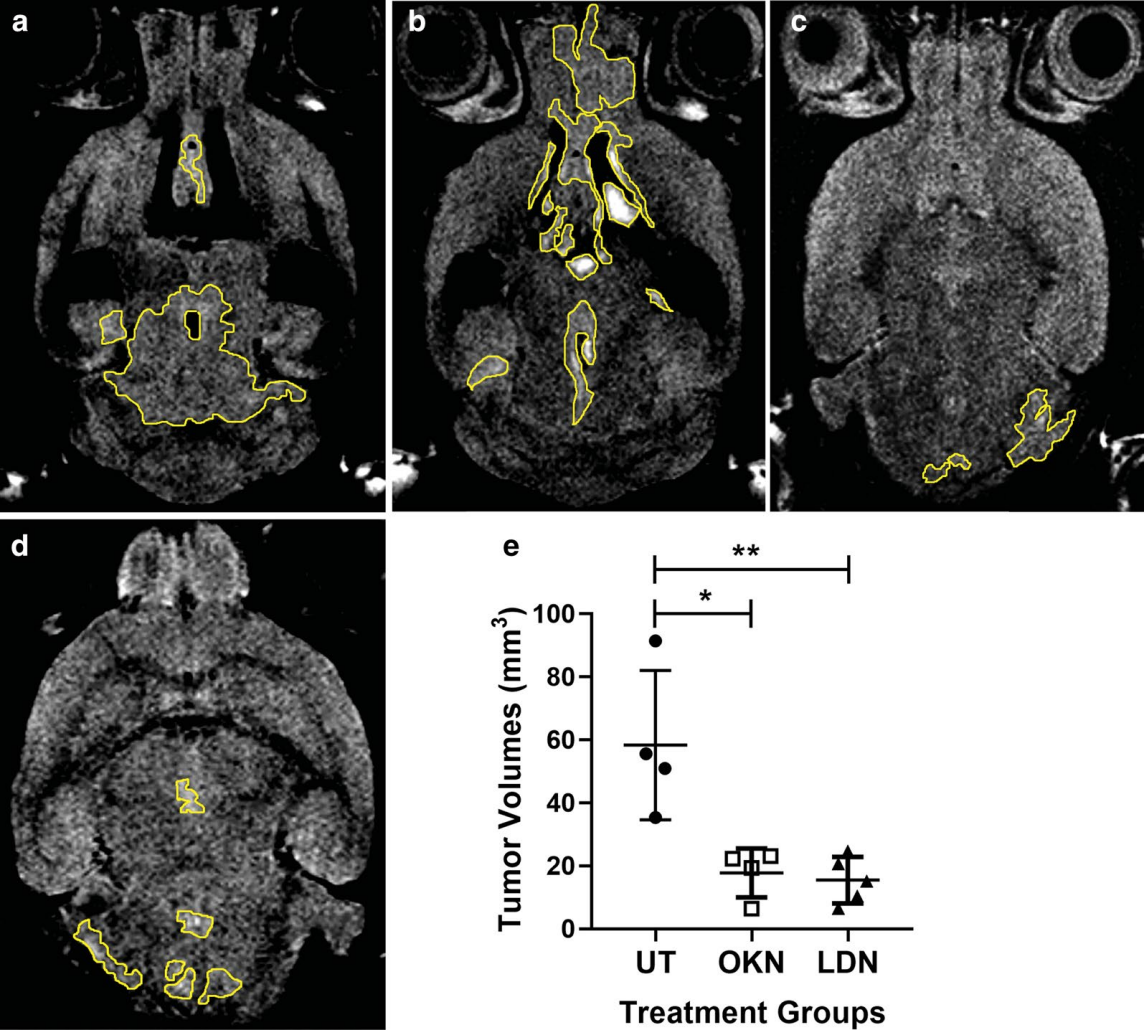
OKN-007 and LDN-193189 both decrease final tumor volumes in a pre-clinical model for DIPG. DIPG final tumor volumes at the completion of treatment. Tumor volumes were computed with additive ROI’s from each slice using FLAIR imaging. **a**, **b** Note the highlighted tumor burden in individual slices for the untreated mice. Figure 1b highlights the potential for leptomeningeal spread to the frontal lobe in this untreated mouse. Tumor burden was visibly reduced in **c** OKN-007 treated, and **d** LDN-193189 treated mice. **e** The final tumor burden in the untreated mice (closed circles; n = 4) was significantly higher than mice treated with both OKN-007 (open squares; n = 4) and LDN-193189 (closed triangles; n = 5). There was no significant difference in final tumor volumes between the two treatment arms. An ANOVA (one-way ANOVA, multiple comparisons) statistical test was used. *p < 0.05 and **p < 0.01

**Fig. 3:**
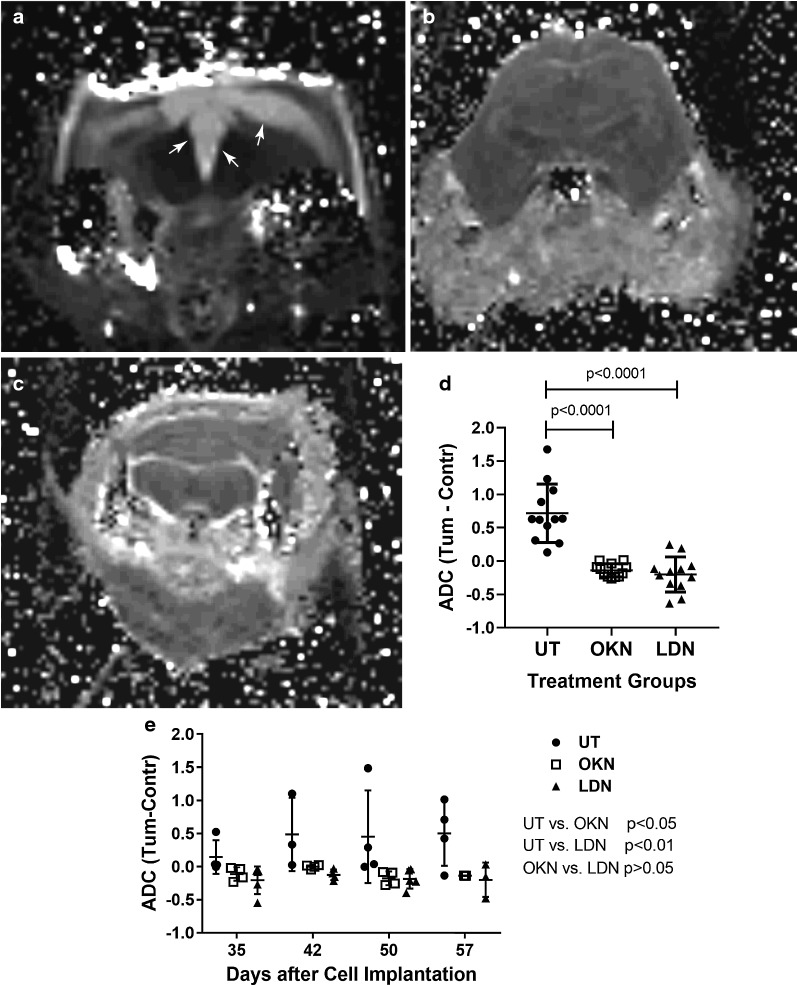
OKN-007 and LDN-193189 both decrease apparent diffusion coefficient (ADC) values in a pre-clinical model for DIPG. ADC values were measured from diffusion-weighted imaging (DWI), at the completion of treatment. **a** The diffusion map for the untreated mouse shows an area of hypointensity/restricted diffusion, likely due to the ventriculomegaly from the tumor burden highlighted with the arrows. This was not seen in mice in either treatment group. **b** Diffusion map of mice treated with OKN-007. **c** Diffusion map of mice treated with LDN-193189. **d** ADC was normalized by subtracting ADC value from the tumor by the ADC value on the contralateral, normal tissue. Untreated mice (closed circles; n = 4; 3 ROIs per sample) had significantly higher ADC values compared to both treatment arms. Although there was no significant difference in ADC between the two treatment arms, both OKN-007 (open squares; n = 4; 3 ROIs per sample) and LDN-193189 (closed triangles; n = 5; 2–3 ROIs per sample) helped normalize the ADC values within the tumor bed. **e** Normalized ADC values obtained at various treatment time-points. OKN-007 and LDN-193189 had significantly lower normalized ADC values compared to untreated tumors (p < 0.05 and p < 0.01, respectively). An ANOVA (one-way ANOVA, multiple comparisons) statistical test was used

MRI assessment is helpful in both preclinical and clinical settings to diagnose and follow response to treatment. Here we describe the MRI characterization of this orthotopic xenograft pediatric DIPG mouse (HSJD-DIPG-007 patient-derived neurospheres) model and the effects of OKN-007 and LDN-193189 to inhibit in vivo pediatric DIPG tumor growth (Figs. 1 and 2). We used conventional T2 scans to identify surgical coordinates, and fluid attenuated inversion recovery (FLAIR) imaging was used to measure tumor volumes for morphological assessment. Gadolinium contrast enhanced images were performed to assure the mice had an intact BBB prior to treatment initiation. Advanced MRI techniques such as diffusion-weighted imaging (DWI) on day 57, after 28 days of treatment, helped characterize water mobility in relation to tumor cellularity and tissue microstructure (Fig. 3). To the best of our knowledge, this study is the first report with MRI characterization as early as 2 weeks post-implantation of an orthotopic xenograft murine model of pediatric DIPG, and the assessment of treatment response using morphological imaging after treatment with OKN-007 and LDN-193189. For this specific study, we did not perform survival analysis because we wanted to compare tumor volumes in untreated, OKN-007, and LDN-193189 treated mice after an equivalent length of treatment.

We hypothesize that mice treated with OKN-007 and LDN-193189 will have decreased final tumor volume compared to the untreated group. The treatment groups included the control group (untreated), OKN-007 treated, and LDN-193189 treated. Therapeutic agents, such as OKN-007 and LDN-193189, were administered at day 28 after surgical implantation of tumor cells. All therapeutic agents were given daily for 28 days, and on day 57, the mice were terminated for immunohistochemistry. With the dismal prognosis and limited effective chemotherapy available for DIPG, there is significant room for continued research studies to help clinicians better understand and treat pediatric DIPG patients. We hope that through our studies in mice, we are able to create hope for children with this devastating disease.

## Methods

### Brain tumor specimen

For the pediatric cells, there was an Institutional Review Board-approved protocol M-1608 from the Hospital Sant Joan de Deu Barcelona. Primary pediatric DIPG cells (HSJD-DIPG-007) were derived from autopsy of a 6-year-old female and were transferred as neurospheres, exponentially growing in suspension in tumorsphere media (TSM) supplemented with corresponding growth factors [15, 16], and checked for mycoplasma contamination.

### Mouse DIPG model

For the animal studies, the Oklahoma Medical Research Foundation Institutional Animal Care and Use Committee approved protocol 18–25. For the DIPG mouse model, HSJD-DIPG-007 neurospheres were implanted into the fourth ventricle of 6–8 week old, male, NOD/SCID (immunocompromised) mice as described previously [14, 15, 17, 18]. All mice were sedated with isoflurane. Each mouse was immobilized using the stereotaxic device and placed on a tooth bar to assure a level, stable field. The surgical field was sterilized using povidone–iodine antiseptic swabs and a 2 cm vertical incision was created. A 1 mm burr hole was drilled after identifying coordinates, 1.4 mm posterior to the lambda and 1.0 mm lateral to midline. A glass gas-tight sterile Hamilton syringe loaded with 5 × 10^5^ DIPG neurospheres mixed with matrigel was suspended in 4 µl volume and further advanced 3.5 mm beneath the skull to reach the fourth ventricle. Cells were injected slowly over the course of 3 min. Following injection, sterile bone cement was applied to the skull and the skin was closed with silk 4.0 surgical sutures. Intraperitoneal buprenex was administered post-operatively for pain relief during recovery. A single dose of intraperitoneal dexamethasone 2 mg/kg was also administered post-operatively to reduce intracerebral edema.

**Fig. 4:**
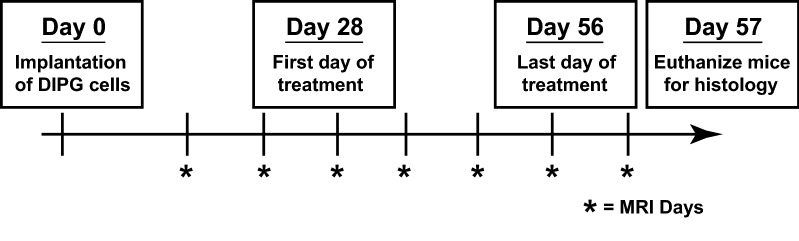
Experimental schema. Day 0 was the day of surgical implantation of patient derived DIPG cells into the fourth ventricle of immunocompromised NOD/SCID mice. Morphological imaging using FLAIR MRI was initiated on day 14 after surgical implantation. MR imaging was continued weekly until termination. Treatment with either OKN-007 or LDN-193189 was initiated on day 28. All treatments were continued for a total of 28 days, with day 56 being the last day of treatments. All mice were euthanized on day 57 when whole brains were extracted for IHC analysis

The experimental schema is depicted in Fig. 4.

### Treatment

The animals were divided into three treatment groups: Untreated (UT) (n = 4), OKN-007 treated (n = 4), LDN-193189 treated (n = 5) mice. Initially, we had planned on having 5 mice in each treatment arm but unfortunately, several mice had to be terminated for neurological deficits noted a few days post-operatively. Both OKN-007 and LDN-193189 treatments were initiated at day 28 after surgery. Treatment with both LDN-193189 and OKN-007 continued daily for 28 days, and mice were terminated for histological analysis on day 58 (see Fig. 4).

OKN-007 (Ryss Laboratories, Union City, CA; 99.8% purity via HPLC) was dissolved in drinking water at a concentration of 150 mg/kg/day, and treatment bottles were changed every 3 days. The optimal concentration of OKN-007 was obtained from a 3 dose in vivo study (unpublished data) in an orthotopic, synegeic GL261 mouse glioma model. Water bottles were weighed prior to bottle changes, and the amount of OKN-007 consumed per mouse was readily determined, as each OKN-007 treated mouse was housed individually. The average consumption of OKN-007 ranged from 130–150 mg/kg/day in all treated mice.

LDN-193189 (Thomas Scientific, Swedesboro, NJ; 98% purity via HPLC) was dosed at 25 mg/kg/day and administered as daily gavage treatments. Dosing was obtained based on the Cavalho et al. [5] study. Plastic tubing oral gavage needles were used. Mice were anesthetized shortly with isoflurane to help decrease agitation during daily gavage treatments.

### Magnetic resonance techniques

#### Morphological imaging

MRI scans were obtained using a 30-cm horizontal Bruker Biospin magnet operating at 7 T (T; Bruker Biospin GmbH, Karsruhe, Germany), with a S116 gradient set to perform all MRI experiments. An EPI (echo planar imaging) transceiver ^1^H 50 W coil with a 38.0 mm inner diameter was used for signal transmission and detection. NOD/SCID mice underwent MR imaging investigations while under anesthesia (isoflurane 1.5–2.0% and 0.8 l/min O_2_) and restrained in a cradle, which was inserted into the MRI scanner. All animals have their body temperature maintained at 37° C with a water bath driven heating pad placed under the animal in the MRI cradle. Respiratory rates were also monitored for the entire duration of the MRI scan. Fluid attenuated inversion recovery (FLAIR) imaging (FOV = 2.0 × 2.0 cm^2^, TR = 10,000 ms, TE = 37.0 ms, matrix size = 256 × 256, averages = 4, slices = 14, slice thickness = 0.7 mm) was used to calculate tumor volumes and to inspect tumor morphology. In-vivo diagnostic contrast-enhanced MRI approach with injection of gadolinium contrast via an intravenous tail-vein catheter was performed at different time intervals to assess for an intact BBB. This assessment used multi-slice spin echo T_1_-weighted images (TR = 1000.0 ms, TE = 14 ms, FOV = 2.50 × 2.50 cm^2^, averages = 2, slices = 16, matrix size = 256 × 256) attained before and 15 min after tail vein gadolinium contrast agent injection (Gd-DTPA, Magnevist, Bayer Inc., Wayne, NY, USA; 0.4 mmol/kg).

MRI scans were initially performed 2 weeks after surgical implantation of tumor cells, followed by weekly imaging until termination (see Fig. 4). During scanning procedures, physiological monitoring for respiration was conducted to assess the animal’s status. Survival of mice following therapeutic protocols were monitored routinely with MRI scans and an MRI were obtained as the last time-point when the mouse is euthanized.

#### Diffusion imaging

Diffusion-weighted imaging (DWI) was also used to characterize the mobility of water in a clinical setting with diffuse tumor involvement. Diffusion data is also valuable in documenting cellular response to drug therapy. A coronal axial multi-slice DWI sequence covering the entire tumor was performed with the following parameters: TR = 3000 ms, TE = 32.5 ms, matrix size = 96 × 96, slice thickness = 0.75 mm. Apparent diffusion coefficient (ADC) values were acquired by placing four circular regions-of-interest (ROIs) within the tumor bed and these values were averaged, and then normalized to the contralateral normal brain.

### Histology and immunohistochemistry

#### Histology staining

Four micron thick histological sections, embedded in paraffin and mounted on HistoBond®Plus slides (Statlab Medical Products, Lewisville, TX) were rehydrated and washed in Tris Buffered Saline (TBS). Rabbit antibodies for anti-Histone H3K27M (cat# 31-1175-00, 1 µg/ml, RevMAB Biosciences, San Francisco, CA), H3K27 tri-methylation (Tri-Methy-Histone H3 (LYS27) (C36B11) Rabbit mAb cat#9733, 1:200, Cell signaling Technology, Danvers, MA), ALK2/ACVR1 (cat# LS-B11761, 1:100, 5 µg/ml, LSBio, Seattle, WA), CD34 (cat# ab81289, 5 µg/ml, abcam, Cambridge, MA), Met (cat# sc-10, 1:50, 4 µg/ml, Santa Cruz Biotechnology, Santa Cruz, CA), Caspase-3 (cat# sc-7148, 1:50, 4 µg/ml, Santa Cruz Biotechnology, Santa Cruz, CA). Nuclear Antigen antibody was raised in mouse against Human (cat# LS-C277289, 1:100, LSBio, Seattle, WA). Slides were processed for Immunohistochemistry using ImmPRESS VR horse anti rabbit IgG Polymer kit (cat# MP-6401, Burlingame, CA) or M.O.M. ImmPRESS™ Peroxidase Polymer kit (cat# MP-2400, Vector Labs Inc., Burlingame, CA). Antigen retrieval (pH 6 Citrate Antigen Unmasking Solution (cat# H-3300, Vector Labs Inc., Burlingame CA) was accomplished via 20 min in a steamer followed by 30 min cooling at room temperature for all antibodies except CD34 which was ten minutes in a steamer followed by 20 min in ice cold pH 6. Sections were treated with a peroxidase blocking reagent (Bloxall, cat# SP-6000, Vector Laboratories, Inc, Burlingame, CA) to inhibit endogenous peroxidase activity, followed by 2.5% normal horse serum or mouse IgG blocking reagent to inhibit nonspecific binding. Appropriate washes were in TBS. Antibodies were applied to each section and following incubation overnight at 4 °C in a humidified chamber, sections were washed in TBS and reagents were applied according to the manufacturer’s directions. Slides were incubated with NovaRed® (Vector Laboratories, Inc., Burlingame, CA) chromogen for visualization. Counterstaining was carried out with Hematoxylin QS Nuclear Counterstain (Vector laboratories, Burlingame, CA). Appropriate positive and negative tissue controls were used.

#### Immunohistochemistry (IHC)

Aperio ScanScope Image Analysis System was used to analyze all the IHCs. H3.K27M, H3.K27-trimethylation (H3.K27me3), ACVR1, and human nuclear antigen IHCs were evaluated using a Positive Pixel Count procedure with the Aperio ImageScope viewer. Although the DIPG were diffuse, five ROIs were placed in heavily stained areas along the brainstem to analyze IHC expression. ROI placement was avoided in areas of necrosis or substantial artifact. The number of positive pixels from ROIs were divided by the total number of pixels (negative and positive) in the examined area, and multiplied by 100, in order to determine the percentage of positivity.

Aperio microvessel analysis algorithm using CD34 as a marker was also utilized to determine microvascular density (MVD, number of vessels per mm^2^). Again, ROIs were arbitrarily selected, and the MVD was calculated for untreated, OKN-007 treated, and LDN-193189 treated groups.

#### Statistics

Graph Pad Prism 8 was the software used for statistical analysis. Statistical significance was assigned only for p values < 0.05. For statistical analysis, ANOVA testing (one-way ANOVA, multiple comparisons) was used for both tumor volume, diffusion data, and IHC analysis to compare the differences of untreated, OKN-007 treated, and LDN-193189 treated pediatric HSJD-DIPG-007 mice. A two-tailed students t-test was used to compare CD34 expression on IHC of OKN-007 and LDN-193189 treated mice.

## Results

The experimental schema is outlined in Fig. 4. Morphological imaging using FLAIR MRI was initiated on day 14 after surgical implantation. MR imaging was continued weekly until termination. Treatment with either OKN-007 or LDN-193189 was initiated on day 28. All treatments were continued for a total of 28 days, with day 56 being the last day of treatments.

**Fig. 5:**
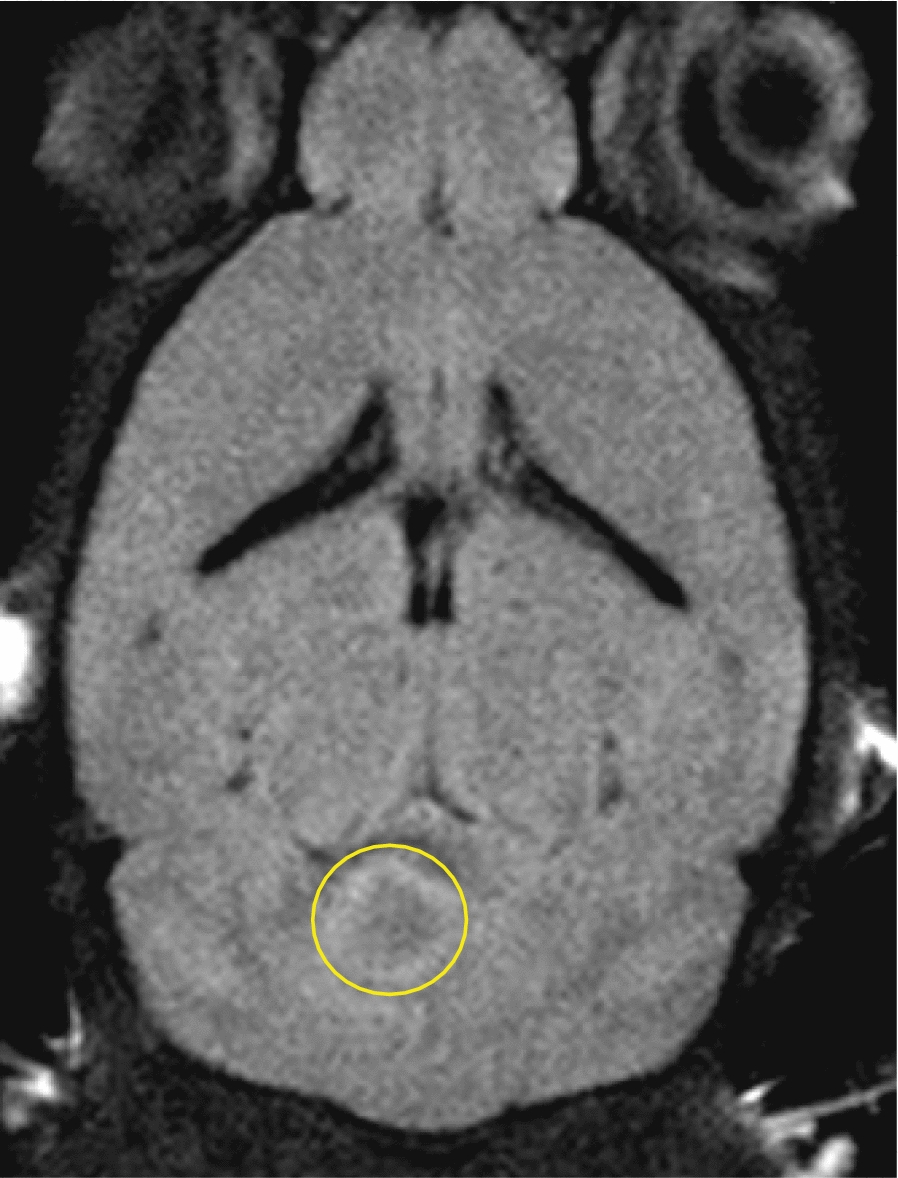
Fluid attenuated inversion recovery (FLAIR) imaging detects DIPG. FLAIR on day 14 after surgical implantation. This image identifies successful tumor engraftment

**Fig. 6:**
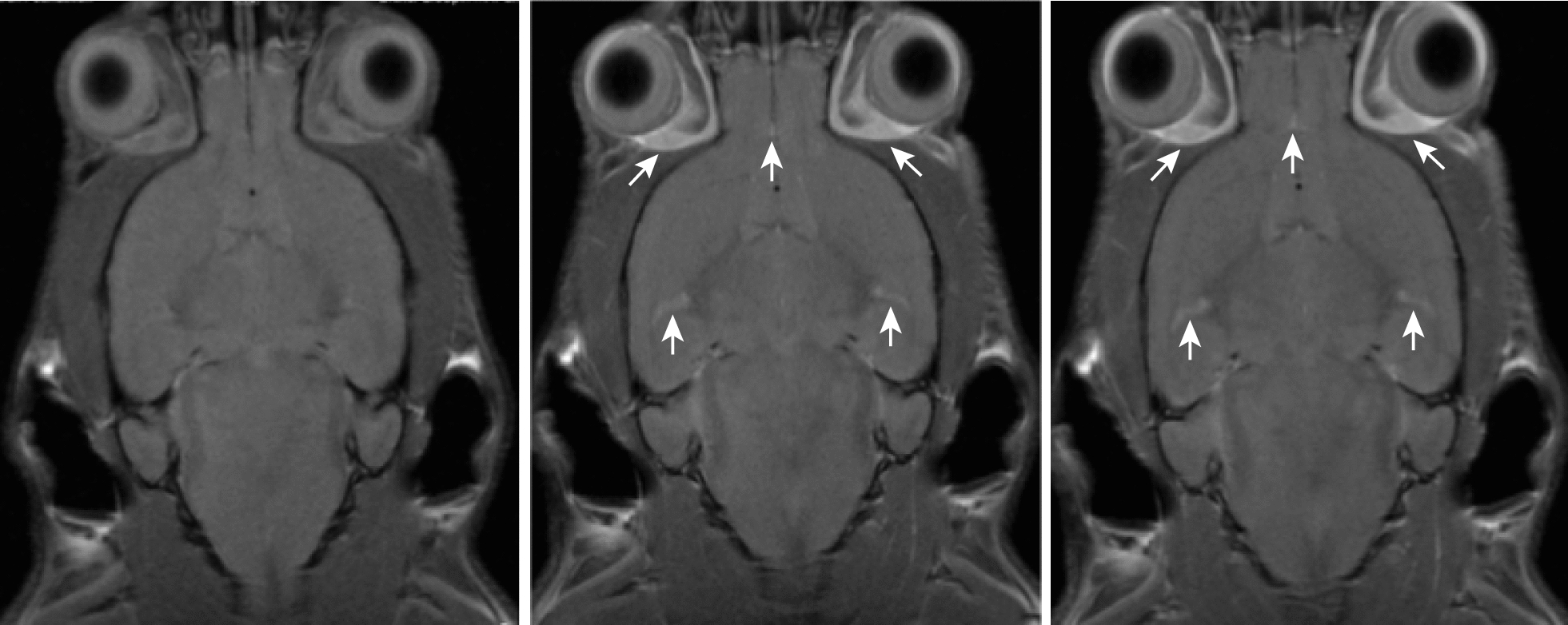
DIPG pre-clinical model emulates intact blood–brain barrier (BBB). T1 contrast enhanced images to evaluate an intact BBB. Gadolinium contrast was injected via tail vein. From left to right, these images were taken pre-contrast, 10 min, and 20 min post contrast injection, respectively. All three images look fairly similar without evidence of increased contrast enhancement in the 10 min and 20 min post contrast images. White arrows depict contrast-enhancement in the eye region and brain vasculature. This supports the presence of an intact BBB, making this an ideal DIPG mouse model

We confirmed tumor engraftment by FLAIR MR imaging as early as 14 days after surgical implantation (Fig. 5). Due to the diffuse nature of DIPG tumors, T2 MR imaging was not able to capture the entirety of the tumor and dismissed tumor involvement in the ventricular space. T2-weighted imaging was only used to confirm coordinates prior to surgical implantation of DIPG cells. All mice underwent FLAIR MR imaging to identify tumor boundaries, T_1_-weighted contrast imaging consistent with the presence of an intact BBB (Fig. 6), DWI, and IHC assessment. Average tumor volumes for each treatment group was assessed weekly using FLAIR imaging. By the end of the treatment plan, tumor volumes stabilized for both OKN-007 and LDN-193189 treated mice, but the untreated mice had a steady incline in tumor volumes with average final tumor burden at approximately 50 mm^3^ (Fig. 1). This tumor burden is significantly larger than both treatment groups that had average tumor volumes of approximately 20 mm^3^ at the end of treatment. Final tumor volumes on day 57 were significantly decreased in the OKN-007 treated mice (16.30 ± 5.17 mm^3^) compared to the untreated mice (32.09 ± 17.63 mm^3^), with p value 0.0013. LDN-193189 also significantly (p value 0.0006) decreased tumor volumes (14.40 ± 3.25 mm^3^) compared to untreated mice. There was no significant difference in final tumor volumes between the two treatment arms (Fig. 2).

The DWI on day 57, prior to termination, showed that the untreated mice had significantly higher (p value < 0.001) normalized apparent diffusion coefficient (ADC) values compared to the normalized ADC values of both treatment groups, OKN-007 and LDN-193189 (Fig. 3). ADC values were normalized by subtracting the ADC within the tumor bed from the ADC within the contralateral normal tissue. The mean normalized ADC values for untreated mice was 0.61 ± 0.42, when mean normalized ADC values was − 0.17 ± 0.21 for OKN-007 treated mice and − 0.17 ± 0.22 for LDN-193189 treated mice. Clearly, both treatment groups had a response to the anticancer therapy based on the ADC ratios compared to the untreated arm. There was no significant difference in the ADC between the two treatment arms. Therefore, OKN-007 has shown to be just as effective as LDN-193189 in decreasing tumor volume and normalizing ADC values.

**Fig. 7:**
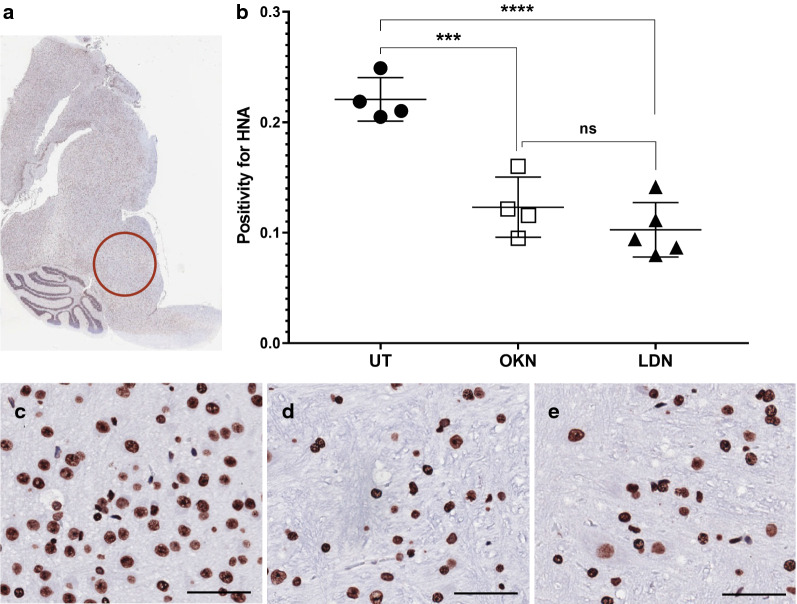
OKN-007 and LDN-193189 both decrease the human nuclear antigen (HNA) in a DIPG model. IHC post-mortem analysis at the completion of treatment, staining for HNA. **a** The ROI’s for data analysis of IHC were placed in the heavily stained regions of the pons as circled in red. **b** Cell stain positivity for anti-human nuclear antigen was significantly higher in the untreated mice compared to both treatment groups. No significant difference was seen between the two treatment arms. Sample legend: Untreated (UT)—closed circles (n = 4); OKN-007 (OKN)—open squares (n = 4); and LDN-193189 (LDN)—closed triangles (n-5). An ANOVA (one-way ANOVA, multiple comparisons) statistical test was used. ***p < 0.001, ****p < 0.0001, and ns is not significant. **c** The 20× view of IHC slides visibly show higher cell stain positivity and cellularity in the untreated mice compared to the **d** OKN-007 treated and **e** LDN-193189 treated mice. The scale bar in each image frame is 100 µm

**Fig. 8:**
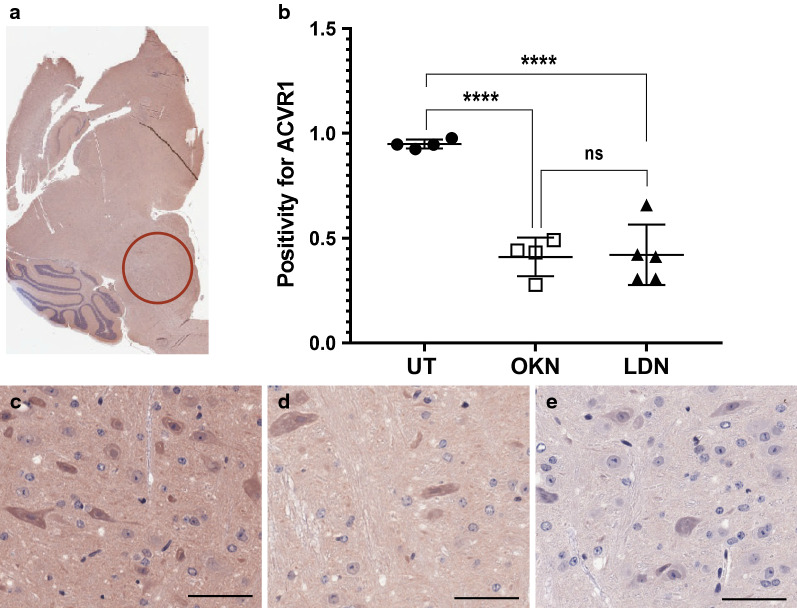
OKN-007 and LDN-193189 both decrease ACVR1 in a DIPG model. IHC analysis staining for ACVR1 antibody. ACVR1 is a common mutation found in DIPG, second to H3 K27M mutation. **a** The ROI’s for data analysis of IHC were placed in the heavily stained regions of the pons as circled in red. **b** Cell stain positivity for ACVR1 antibody was higher in the untreated mice compared to both treatment groups. There was no difference between the two treatment arms. Sample legend: Untreated (UT)—closed circles (n = 4); OKN-007 (OKN)—open squares (n = 4); and LDN-193189 (LDN)—closed triangles (n-5). An ANOVA (one-way ANOVA, multiple comparisons) statistical test was used. ****p < 0.0001, and ns is not significant. **c** The 20× view of IHC slides visibly show higher cell stain positivity for ACVR1 in the untreated mice compared to the **d** OKN-007 treated and **e** LDN-193189 treated mice. The scale bar in each image frame is 100 µm

**Fig. 9:**
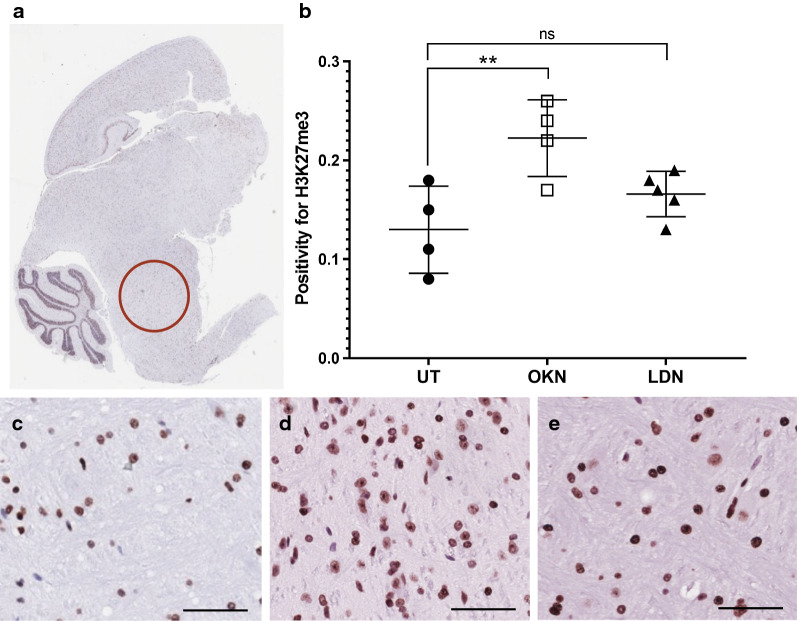
OKN-007 increases H3.K27me3 expression, better than LDN-193189, in a DIPG model. IHC analysis staining for H3.K27me3. **a** The ROI’s for data analysis using IHC were placed in the heavily stained regions of the pons as circled in red. **b** H3.K27me3 is a marker of H3.K27M mutation post-translational modification and our results show that treatment with OKN-007 significantly increased H3.K27me3 expression compared to untreated mice or LDN-193189-treated mice. Sample legend: Untreated (UT)—closed circles (n = 4); OKN-007 (OKN)—open squares (n = 4); and LDN-193189 (LDN)—closed triangles (n-5). An ANOVA (one-way ANOVA, multiple comparisons) statistical test was used. *p < 0.05, **p < 0.01 and ***p < 0.001. **c** The 20× view of IHC slides visibly show the lowest H3.K27me3 expression in the untreated mice compared to the **d** OKN-007 treated mice. There was no significant difference between **e** LDN-193189 treated mice and untreated mice. The scale bar in each image frame is 100 µm

**Fig. 10:**
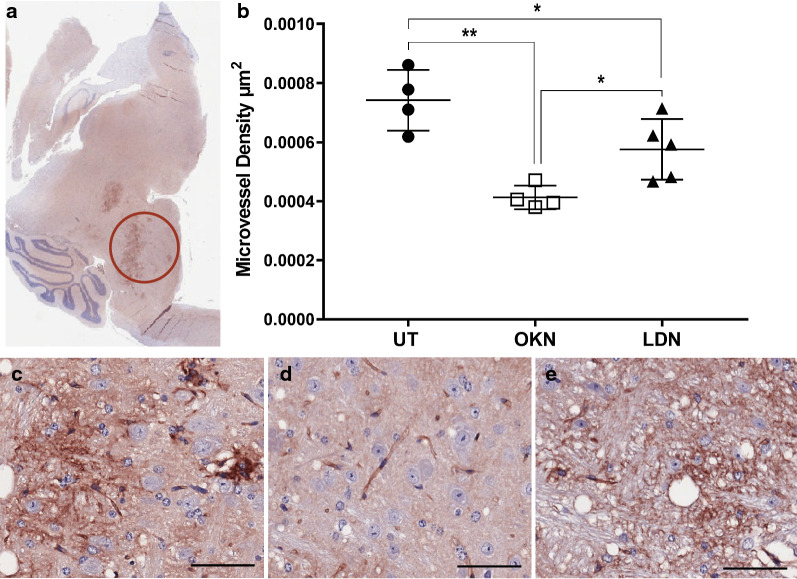
OKN-007 decreases micro-vessel density (MVD) better than LDN-193189 in a DIPG model. IHC analysis staining for CD34 expression, a marker for MVD. **a** The ROI’s for data analysis of IHC were placed in the heavily stained regions of the pons as circled in red. **b** CD34 expression was significantly higher in the untreated mice compared to both treatment arms when using ANOVA multivariate testing. There was also a significant decrease in CD34 expression in the OKN-007 treated mice compared to LDN-193189 treated mice, when using a t-test for statistical analysis. Sample legend: Untreated (UT)—closed circles (n = 4); OKN-007 (OKN)—open squares (n = 4); and LDN-193189 (LDN)—closed triangles (n-5). An ANOVA (one-way ANOVA, multiple comparisons) statistical test was used. *p < 0.05 and **p < 0.01. **c** The 20× view of IHC slides visibly show increased microvascular density in the untreated mice compared to the **d** OKN-007 treated and **e** LDN-193189 treated mice. The scale bar in each image frame is 100 µm

**Fig. 11:**
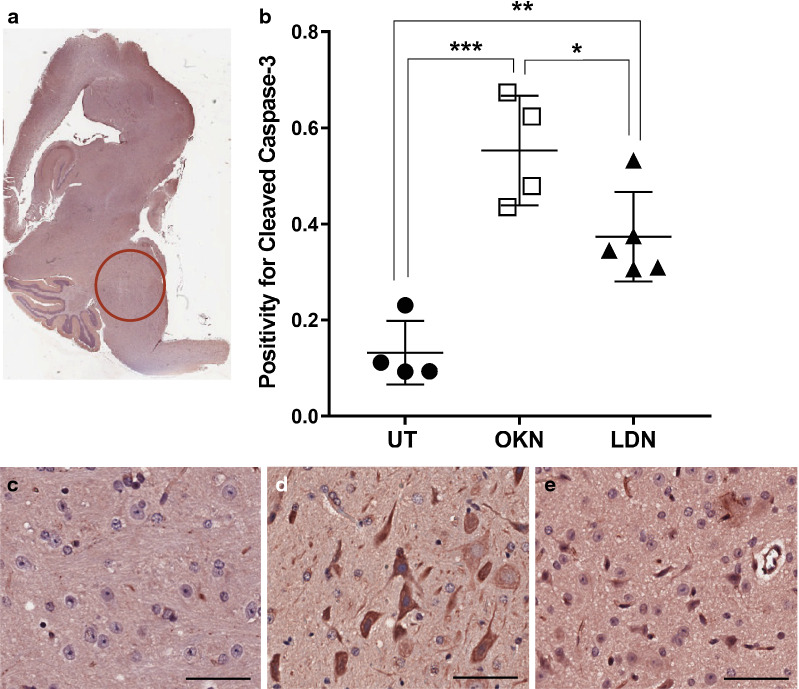
OKN-007 increases apoptosis, better than LDN-193189, in a DIPG model. IHC analysis staining for cleaved caspase-3. **a** The ROI’s for data analysis using IHC were placed in the heavily stained regions of the pons as circled in red. **b** Cleaved caspase-3 is a marker of apoptosis and our results show that treatment with OKN-007 and LDN-193189 significantly increased cleaved caspase-3 expression compared to untreated mice. Mice treated with OKN-007 had the highest cleaved caspase-3 activity. Sample legend: Untreated (UT)—closed circles (n = 4); OKN-007 (OKN)—open squares (n = 4); and LDN-193189 (LDN)—closed triangles (n-5). An ANOVA (one-way ANOVA, multiple comparisons) statistical test was used. *p < 0.05, **p < 0.01 and ***p < 0.001. **c** The 20× view of IHC slides visibly show the lowest cleaved caspase-3 expression in the untreated mice compared to the **d** OKN-007 treated and **e** LDN-193189 treated mice. The scale bar in each image frame is 100 µm

**Fig. 12:**
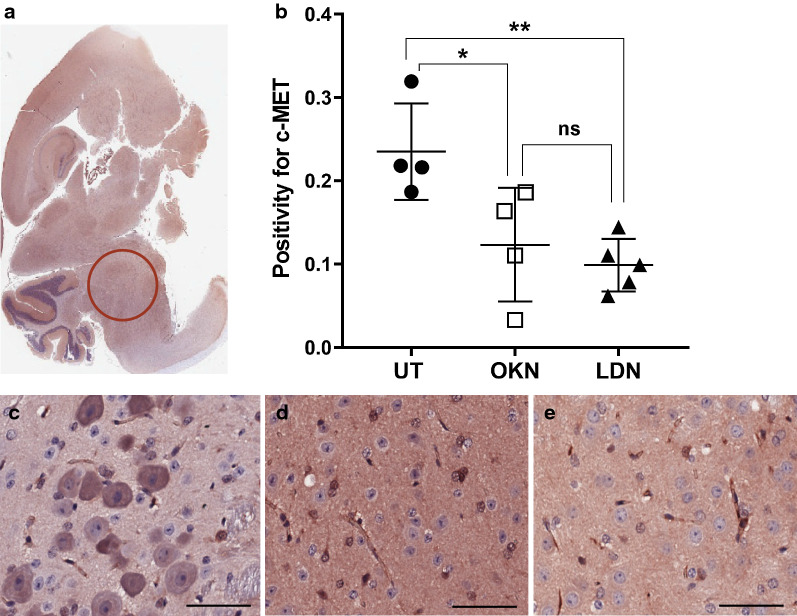
OKN-007 and LDN-193189 both decrease c-MET in a DIPG model. IHC analysis staining for c-MET. c-MET is highly expressed in half of DIPG cases. **a** The ROI’s for data analysis of IHC were placed in the heavily stained regions of the pons as circled in red. **b** Data suggests that untreated mice had significantly higher c-MET expression compared to both treatment groups. There was no significant difference in c-MET expression between the OKN-007 and LDN-193189 treated mice. Sample legend: Untreated (UT)—closed circles (n = 4); OKN-007 (OKN)—open squares (n = 4); and LDN-193189 (LDN)—closed triangles (n-5). An ANOVA (one-way ANOVA, multiple comparisons) statistical test was used. *p < 0.05, **p < 0.01, and ns is not significant. **c** The 20× view of IHC slides visibly show higher cell stain positivity for c-MET in the untreated mice compared to the **d** OKN-007 treated and **e** LDN-193189 treated mice. The scale bar in each image frame is 100 µm

IHC analysis was performed for human nuclear antigen, ACVR1 antibody, CD34, H3.K27M, H3.K27me3, cleaved caspase-3, and c-met. Cell stain positivity for anti-human nuclear antigen expression was significantly decreased in both treatment groups compared to the untreated arm (0.22 ± 0.02). This analysis showed p value of 0.0005 when comparing OKN-007 treated (0.12 ± 0.03) and untreated mice, and a p value < 0.0001 when comparing LDN-193189 treated (0.10 ± 0.03) and untreated mice (Fig. 7). There was no statistical difference between the two treatment arms. Similarly, ACVR1 expression was significantly decreased in the treatment groups compared to the untreated mice (0.95 ± 0.02), with a p value < 0.0001 when both treatment groups were compared to the untreated arm (Fig. 8). Although treatment with LDN-193189 was expected to decrease ACVR1 expression (0.42 ± 0.14) as it is a known ACVR1 inhibitor, OKN-007 also significantly reduced ACVR1 (0.41 ± 0.09). IHC analysis of H3.K27M expression was not statistically different between the untreated mice and the treatment groups, and therefore data was not presented. However, H3.K27me3 expression indicated a significant increase in positivity following OKN-007 treatment (0.22 ± 0.04) compared to untreated mice (0.13 ± 0.04; p < 0.01) (Fig. 9). IHC was also used to quantify CD34 expression, a good marker for microvascular density and measure of angiogenesis. CD34 expression was significantly decreased in the treatment groups compared to the untreated mice (0.00076 ± 0.00014), with a p value of 0.001 for OKN-007 (0.00044 ± 0.00008) vs. untreated mice and a p value of 0.0457 for LDN-193189 (0.00057 ± 0.00017) vs. untreated mice. There was also a significant decrease in CD34 expression in the OKN-007 treated mice compared to LDN-193189 treated mice, with a p value of 0.0212 (Fig. 10). IHC analysis of cleaved caspase-3 was also performed to compare the level of apoptosis in the treated vs. untreated mice. OKN-007 treated mice had significantly higher expression of cleaved caspase-3 (0.55 ± 0.11) compared to both untreated mice (0.13 ± 0.07; p value 0.0002) and LDN-193189 treated mice (0.37 ± 0.09; p value 0.0405). LDN-193189 treated mice also had more cleaved caspase-3 cell stain positivity compared to untreated mice (p value 0.008), and is a mechanism of action not previously described for this agent (Fig. 11). Lastly, c-MET expression was also evaluated by IHC as increased c-MET aids in tumor progression and proliferation. c-MET is one of many HIF-1α transcriptional targets, as well as GLUT1 (glucose transporter 1), BNIP3 (BCL2/adenovirus E1B 19 kDa protein-interacting protein 3) and erythropoietin [19]. Untreated mice had significantly higher c-MET expression (0.24 ± 0.06) compared to both the OKN-007 treated mice (0.12 ± 0.07; p value of 0.0334) and LDN-193189 treated mice (0.09 ± 0.03; p value of 0.0084) (Fig. 12). IHC analysis has confirmed the molecular benefit of treatment with OKN-007. Treatment with OKN-007 has been equally effective to LDN-193189 in decreasing expression of human nuclear antigen, ACVR1 antibody, and c-MET. OKN-007 has the added benefit of decreasing angiogenesis by suppressing CD34 expression, and also promotes apoptosis by increasing cleaved caspase-3 expression compared to LDN-193189 treated mice. Our data using OKN-007 is promising and future studies in vivo may further substantiate its use to prevent DIPG tumor angiogenesis and progression.

## Discussion

DIPG is an aggressive and infiltrative pontine tumor that carries a grim prognosis, accounting for the majority of deaths secondary to pediatric brain tumors [20]. Considering that radiation therapy has only shown palliative benefits and no significant improvement has been noted with the addition of adjuvant chemotherapy, there is significant room for therapeutic advancements to treat pediatric DIPG [2, 21].

### Magnetic resonance imaging

Historically, patients with DIPG were diagnosed with clinical symptoms and MRI findings alone. Diagnosis by biopsy findings is novel and prevalent in only large clinical centers [2, 22]. Nonetheless, MR imaging continues to be vital for diagnosis and follow-up of therapeutic response. We describe the first pre-clinical DIPG mouse model with serial assessment of tumor growth from 2 weeks post-surgical implantation to 57 days post-implantation to evaluate tumor growth/regression with therapeutic agents. Prior pre-clinical DIPG mouse models used bioluminescence and only characterized DIPG tumors in mice by MRI 78 days after implantation [15].

FLAIR MR imaging demonstrated tumor growth as early as 2 weeks post-implantation and is conventionally the MR imaging modality of choice in clinical settings. T2-weighted morphological imaging had been used for previous glioma models to measure tumor volumes; however, DIPG created more diffuse tumors with unclear boundaries and tumors also invade the ventricular space [6, 11]. Tumors within the ventricular space cannot be recognized using T2-weighted imaging as cerebrospinal fluid (CSF) has higher intensity signaling. FLAIR MR imaging offers the advantage of suppressing the signal intensity of CSF fluid and accentuating periventricular lesions [23]. The use of FLAIR imaging has assisted us in identifying tumor boundaries within the pontine region as well as metastatic sites. Literature suggests that one-third of DIPG patients have leptomeningeal spread of their tumor [24]. We were able to identify frontal lobe involvement of the DIPG tumors in a few mice using FLAIR imaging.

Another valuable imaging tool is diffusion weighted imaging (DWI) which uses the movement of water molecules to produce contrast in MR images. DIPG tumors are infiltrative and diffuse in nature, resulting in defective cell membranes and more inflammation, therefore resulting in the increased motion of water molecules extracellularly [25]. This is different from what would be expected in solid tumors with increased cellularity and movement restriction of water molecules. Within DIPG, there are prognostically variable subsets that may be identified using apparent diffusion coefficient (ADC) values derived from DWI. A low ADC value (< 1300 × 10^–6^ mm^2^/s) was shown to have median survival of 3 months with tumor tissue consistent with high-grade histology. On the contrary, higher ADC values (> 1300 × 10^–6^ mm^2^/s) are associated with median survival of 13 months and low-grade histology with H3.1 K27M mutation [26]. The HSJD-DIPG-007 patient-derived neurospheres that we used to create a preclinical DIPG mouse model were found to have mean ADC value of 611 × 10^–6^ mm^2^/s in the untreated group. This is consistent with the study that shows high-grade histology DIPG tumors having a lower ADC value, as the HSJD-DIPG-007 neurospheres have both the H3.3 K27M mutation and the ACVR1 mutation [18, 27].

DWI can also be used to monitor treatment response. After 28 days of treatment with both OKN-007 and LDN-193189, the normalized ADC values were significantly lower (p value < 0.0001) than the untreated arm. Although there was no significant difference between the two treatment arms, there was notable treatment response using both these agents. Lower ADC values in response to treatment suggests that both OKN-007 and LDN-193189 helped create an environment with less inflammation, fewer DIPG cells, and more normal brain cells with intact cell membranes.

It is likely that advanced disease, which is the case when most patients present with symptoms, results in partial disruption of the BBB especially at the microscopic level. When creating a DIPG model, we wanted to assure that the BBB was intact prior to initiating treatment, as would be the case on early identification of the disease, and this characteristic is one of the major challenges of DIPG treatment. We performed T_1_-weighted MR imaging before and after injection of gadolinium contrast via tail vein on day 28, prior to treatment initiation. T_1_-weighted images pre-contrast, followed by 5, 10, 15, and 20 min post-contrast showed no significant difference in signal intensity, consistent with an intact BBB. Our study first confirmed tumor engraftment by FLAIR imaging and then assured that the mice had a relatively intact BBB at day 28, prior to treatment initiation.

### Treatment

Radiotherapy is routinely used to treat DIPG, but unfortunately is palliative in nature. Prior studies have not demonstrated any survival benefit with the use of adjuvant chemotherapy versus radiation therapy alone [19, 21]. The ineffective response to chemotherapeutic agents alone is likely due to the intact BBB seen in DIPG [28]. This is a significant challenge in therapeutic development and the primary reason we chose to treat DIPG mouse models with OKN-007. OKN-007 is a small molecule that crosses the BBB. Coutinho de Souza et al. describes the high performance liquid chromatography-derived brain tissue levels of OKN-007 treated rats and demonstrated that OKN-007 was taken up by both normal brain and tumor tissue, equally, following oral administration of OKN-007 [11]. This data identifies a true strength of OKN-007 to be used alone and possibly also with adjuvant chemotherapy.

The potential of OKN-007 as an anti-cancer agent is due to its ability to decrease cell proliferation and angiogenesis [9, 29]. OKN-007 decreases angiogenesis by inhibition of both HIF-1 protein expression and vascular endothelial growth factor receptor 2 (VEGFR2) protein expression [9, 12]. Our lab has also previously identified the ability of OKN-007 to increase apoptosis [9]. Our group also previously assessed tumor rebound in a rat C6 glioma model, which indicated that OKN-007-treated tumors did not return for over 60 days, after a 10 day treatment period [8]. There are continued efforts to understand the primary mechanism of action of OKN-007, but its antiangiogenic and pro-apoptotic properties render it a valuable therapeutic agent for DIPG.

In addition to OKN-007, we also evaluated the efficacy of LDN-193189 for the treatment of pediatric DIPG. LDN-193189 is a small molecule derived from dorsomorphin, an inhibitor of the BMP pathway. It has been effective in decreasing cellular viability in multiple human cancer cell lines including glioblastoma, breast, lung, pancreatic, cervical, and prostate cancers [30]. Unfortunately, despite its potent and selective inhibition of the BMP pathway, it is a multi-kinase inhibitor with low specificity as it inhibits a wide range of receptors and intracellular kinases. When tested against a panel of protein kinases, even at the lowest concentration of 0.1 µM of LDN-193189, it potently inhibited RIPK2, FGF-R1, NUAK1, MINK1, GCK, VEG-FR, MAPK8, AKT. It is likely that LDN-193189 may also inhibit other kinases not yet evaluated [31]. Therefore, LDN-193189 is likely not an ideal molecule for DIPG treatment due to the high side effect profile expected with potent inhibition of numerous protein kinase pathways. Prior studies using LDN-193189 also reported weight loss as a side effect [5].

This study revealed the beneficial effects of OKN-007 in a preclinical mouse model of DIPG using both MRI characteristics and immunohistochemistry. Treatment with both OKN-007 and LDN-193189 significantly decreased tumor volumes and ADC values compared to the untreated group. Our study confirmed the pre-clinical benefits of LDN-193189, as previously reported. A high side-effect profile is expected as LDN-193189 is a multi-kinase inhibitor [30, 31]. The preclinical effects of OKN-007 on decreasing tumor volume in this xenograft DIPG model with a favorable safety profile, as previously reported in human stroke trials, is promising [32]. Not only has OKN-007 shown preclinical efficacy against liver cancer, colon cancer and brain tumors such as glioblastoma, but it has clear potential as a therapeutic agent against pediatric DIPG [23, 33, 34]. OKN-007 would be a promising treatment to consider in combination with involved field radiation to potentiate the effects of radiation. The safety profile of OKN-007 is potentially higher than chemotherapeutic agents that are used in conjunction with involved field radiation.

This study focused on treatment effects comparing a similar time-frame for all treatment groups. A survival study will be incorporated in future studies.

### Immunohistochemistry

IHC was also used to confirm cellular response to treatment. Treatment groups displayed a significant decrease in human nuclear antigen, ACVR1, CD34, and c-MET expression compared to the untreated mice. We had expected a decrease in ACVR1 expression in LDN-193189 treated mice as this is its sole mechanism of action, but we were pleased to see similar protein suppression in OKN-007 treated mice. OKN-007 treated mice also had significantly decreased CD34 expression compared to both the untreated and LDN-193189 treated mice, making this a promising therapeutic anti-cancer agent. Unexpectedly, we also found that OKN-007 increased H3.K27me3 levels, whereas LDN-193189 did not, compared to untreated tumors. Huang et al. [35] previously indicated decreased H3.K27me3 in H3.K27M mutant tumors, compared to wild-type, and that loss of H3.K27me3 is associated with poorer overall survival, as well as a greater likelihood of disease progression in DIPG patients. CD34 is a marker of microvascular density and angiogenesis, which is vital for tumor progression. Increased CD34 expression has been associated with new blood vessel growth and has been found in high-grade gliomas [36, 37]. OKN-007 favorably decreased expression of CD34, and may be considered a useful targeted therapy to improve overall prognosis. Along with common mutations such as ACVR1 and H3 K27M found in DIPG, 50% of DIPG cases harbor gain of function processes that stimulate tyrosine kinase receptors to trigger further tumor progression [2]. Increased c-MET, a known hepatocyte growth factor receptor, is a similar component found in DIPG that we tested using IHC. As expected, c-MET expression was higher in untreated mice, while both treatment arms appropriately decreased levels of c-MET.

Cleaved caspase-3 is often used to evaluate the presence of apoptosis. Decreased caspase-3 activation usually signifies less apoptosis and unopposed cell proliferation, which promotes further tumor growth [38]. Increased cleaved caspase-3 expression shifts the balance of apoptosis vs. cell proliferation to favor more apoptosis, and therefore inhibit tumor growth [39]. Our results show that both treatment arms had higher expression of cleaved caspase-3 compared to the untreated arm. OKN-007 had a significantly higher level of apoptosis even compared to LDN-193189 treated mice and should be considered a therapeutic agent by itself.

## Conclusions

With the dismal prognosis and limited effective chemotherapy available for DIPG, there is significant room for continued research studies and OKN-007 merits further exploration as a therapeutic agent. We particularly found that this agent, which readily crosses the BBB, can significantly decrease tumor volumes, ADC values, MVD, and HNA, c-MET and ACVR1 protein expressions, compared to untreated tumors, as well as significantly increase apoptosis. In addition, OKN-007 was able to perform better than an ACVR1 inhibitor in reducing MVD, and increasing apoptosis or H3.K27me3.

## Data Availability

The datasets used and/or analysed during the current study are available from the corresponding author on reasonable request.

## Abbreviations

ACVR1: Activin A receptor, type I
ADC: Apparent diffusion coefficient
BBB: Blood brain barrier
DIPG: Diffuse intrinsic pontine glioma
DWI: Diffusion-weighted imaging
FLAIR: Fluid attenuated inversion recovery
GLUT-1: Glucose transporter 1
H3.K27me3: H3.K27 tri-methylation
HIF-1α: Hypoxia inducible factor 1 α
HNA: Human nuclear antigen
IHC: Immunohistochemistry
MRI: Magnetic resonance imaging
MVD: Microvessel density
OKN-007: OKlahoma Nitrone-007
PDX: Patient-derived xenograft
SULF2: Sulfatase 2
TGFβ1: Transforming growth factor β1
TSM: Tumorsphere media
UT: Untreated
VEGFR2: Vascular endothelial growth factor receptor 2
